# Natural aluminosilicate nanotubes loaded with RuCo as nanoreactors for Fischer-Tropsch synthesis

**DOI:** 10.1080/14686996.2021.2017754

**Published:** 2022-01-18

**Authors:** Kristina Mazurova, Aleksandr Glotov, Mikhail Kotelev, Oleg Eliseev, Pavel Gushchin, Maria Rubtsova, Anna Vutolkina, Ruslan Kazantsev, Vladimir Vinokurov, Anna Stavitskaya

**Affiliations:** a Department of Physical and Colloid Chemistry, Gubkin University, Moscow, Russia; b Laboratory of Catalytic Reactions of Carbon Oxides, N.d. Zelinsky Institute of Organic Chemistry, RAS, Moscow, Russia; c Chemical Department, Moscow State University, Moscow, Russia

**Keywords:** Halloysite, сobalt, ruthenium, Fischer–Tropsch, carbon monoxide, hydrogen, 102 Porous/Nanoporous/Nanostructured materials <, 100 Materials, 205 Catalyst/Photocatalyst/Photosynthesis <, 200 Applications

## Abstract

Following nanoarchitectural approach, mesoporous halloysite nanotubes with internal surface composed of alumina were loaded with 5–6 nm RuCo nanoparticles by sequential loading/reduction procedure. Ruthenium nanoclusters were loaded inside clay tube by microwave-assisted method followed by cobalt ions electrostatic attraction to ruthenium during wetness impregnation step. Developed nanoreactors with bimetallic RuCo nanoparticles were investigated as catalysts for the Fischer-Tropsch process. The catalyst with 14.3 wt.% of Co and 0.15 wt.% of Ru showed high activity (СO conversion reached 24.6%), low selectivity to methane (11.9%), CO_2_ (0.3%), selectivity to C_5+_ hydrocarbons of 79.1% and chain growth index (α) = 0.853. Proposed nanoreactors showed better selectivity to target products combined with high activity in comparison to the similar bimetallic systems supported on synthetic porous materials. It was shown that reducing agent (NaBH_4_ or H_2_) used to obtain Ru nanoclusters at first synthesis step played a very important role in the reducibility and selectivity of resulting RuCo catalysts.

## GRAPHICAL ABSTRACT

**Figure:**
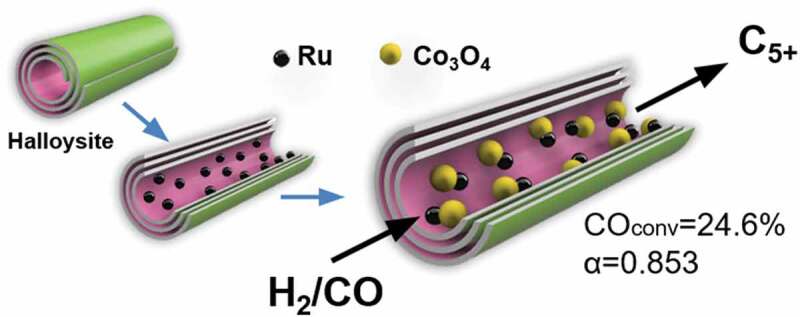

## 1. Introduction

The Fischer-Tropsch process is a sustainable industrial process that opens new possibilities for conversion of waste to valuable products. Conversion of natural gas, coal, carbon dioxide and monoxide, biomass and even plastic to high-quality synthetic fuels, waxes, lubricants, monomers, oxygenates is possible using the Fischer-Tropsch synthesis (FTS) [1–3]. The yield and distribution of Fischer-Tropsch synthesis products can be controlled both by operating conditions (pressure, temperature, gas hourly space velocity) and composition of a catalyst [4]. Supported cobalt catalysts are widely applied in industry to synthesize liquid C_5+_ hydrocarbons due to their high catalytic activity, selectivity and low activity in the water gas shift reaction [5–9].

It is a common knowledge that nature of a support has a significant effect on the activity and selectivity of FTS catalysts [10–15]. Among various supports, nanotubes are very perspective due to the ability to include active particles inside and prevent their aggregation. For example, loading of metal particles inside carbon nanotubes (CNTs) led to an increase in the efficiency of the FT process [16–18]. However, the regeneration of such systems by burning out coke is impossible. This significantly complicates application of Co@CNTs on an industrial scale. Another positive feature of halloysite clay nanotubes is their biological and ecological biocompatibility. On the examples of heavy metal containing nanoparticles, it was shown that such systems possessed no acute toxicity to model cells and organisms [19,20].

Halloysite – a mesoporous multiwalled aluminosilicate nanotubes with an outer diameter of 50–60 nm, an inner lumen diameter of 10–20 nm, a distance between the walls of 40 nm and a length from 500 nm to 1.5 μm – is proposed here as a new support for RuCo FT catalysts. The outer surface of nanotubes consists of silica, while the inner surface is made of alumina [21]. Tubular structure with possibilities for modification combined with thermal and mechanical stability, low acidity of halloysite make it an interesting support for FTS catalysts [22,23]. As the most active catalysts are those with 8–9 nm Co nanoparticles [24,25], the mesoporous lumen of halloysite is a perfect place for Co nanoparticles with the favourable size. Hence, no study has been made on the investigation of cobalt catalysts promoted with ruthenium-based on this tubular clay. Ruthenium is the most active promoter in the row of noble metals that acts as a structural additive for FT cobalt catalysts. It prevents agglomeration of cobalt oxide particles during calcination and leads to an increase in the selectivity towards long-chain n-paraffins [13,26–28]. The addition of Ru also inhibits the deactivation of cobalt catalysts, catalysing the hydrogenolysis of carbonaceous deposits [24]. Together with the influence of the promoting agent and the nature of the support, methods of catalyst preparation may tune the activity and selectivity [25–30]. The wetness impregnation method is the most common for preparing supported metal catalysts [31]. The order of impregnation in bimetallic catalyst plays a decisive role in a Ru-promoted cobalt-based catalyst. The hybrid ‘reduction-impregnation’ method was shown to be more efficient in comparison with co-impregnation and sequential reduction [32].

In this work, natural aluminosilicate nanotubes (halloysite) were studied as nanocontainers for bimetallic RuCo nanoparticles. The main objectives were to develop a synthesis procedure to obtain bimetallic RuCo nanoparticles inside clay nanotubes and study these core-shell systems in FTS. Hybrid microwaves-assisted reduction–impregnation method was proposed to achieve halloysite tubes loading with metals. Together with the influence of Ru promotion, the effect of reducing agent (NaBH_4_ or H_2_) on reducibility and efficiency of catalysts was studied. It was shown that reducing procedure played a very important role in the reducibility and selectivity of RuCo catalysts.

## 2. Experimental details

### 2.1. Materials

Aluminosilicate nanotubes halloysite (HNT) (Al_2_Si_2_O_5_(OH)_4_) (Sigma-Aldrich), cobalt nitrate hexahydrate (Сo(NO_3_)_2_·6H_2_O) (RusChem), ruthenium chloride (RuCl_3_) (Aurat), sodium borohydride powder (NaBH_4_) (RusChem) were used.

### 2.2. Catalysts preparation

For the synthesis of Co-encapsulated halloysite nanotubes (**Co@HNT**), cobalt nitrate hexahydrate (Co(NO_3_)_2_·6H_2_O) was dissolved in distilled water (5 mL) at a metal concentration of 15% based on the weight of the support. Halloysite (1 g) was impregnated by incipient wetness impregnation of 0.3–0.5 ml of the resulting solution and dried at 80°C, and the process was repeated until the metal was completely precipitated. At the last stage of the synthesis, the sample was completely dried and calcined in air at 350°C for 4 hours.

Halloysite (1 g) and ruthenium chloride (5 mg) were dispersed in ethyl alcohol (30 mL) by sonication for 30 minutes. To intercalate ruthenium nanoparticles into the inner cavity of aluminosilicate nanotubes, the resulting suspension was treated in a microwave oven for 10 minutes. The mixture was then centrifuged (6500 rpm, 3 minutes), washed, dried at 65°C for 24 hours, and reduced with hydrogen (8%vol.) diluted in argon. At the second stage, the sample was impregnated with an aqueous solution of cobalt nitrate by the incipient wetness impregnation method similar to the preparation of Co/HNT. As a result, **RuCo@HNT-1** was obtained.

To obtain **RuCo@HNT-2**, the same procedure was carried out, but instead of hydrogen, an aqueous solution of sodium borohydride (NaBH_4_) (0.5M) was used as a reducing agent. The reduced sample was washed three times to remove by-products and dried at 65°C for 24 hours. At the second stage of the synthesis, the sample was impregnated with an aqueous solution of cobalt nitrate by the incipient wetness impregnation method similar to the preparation of Co/HNT.

### 2.3. Catalysts characterization

Textural characteristics were determined using low-temperature nitrogen adsorption-desorption on a Micromeritics Gemini VII 2390 t instrument (USA). Before measurements, the samples were degassed at a temperature of 300°C for 4 hours. Based on the results of adsorption in the range of relative pressures P/P_0_ = 0.05–0.35, the specific surface area of the prepared catalysts was calculated by the Brunauer – Emmett – Teller (BET) method, pore volume, and diameter were obtained from the Barrett – Joyner – Halenda model.

The programmed hydrogen reduction (TPR) was performed on a Micromeritics AutoChem HP2950 instrument (USA). Before measurements, the samples were degassed at a temperature of 800°C for 3 hours. The sample (0.1 g) was placed in a quartz reactor and purged with argon flow (rate of 30 mL/min) at 50°C for 1 hour. The reduction was carried out in a H_2_ + Ar gas mixture (H_2_ = 8%vol., Ar-balance) with a flow rate of 30 mL/min. To obtain the TPR curve, the temperature was gradually raised to 800°C with a heating rate of 10°C/min.

The morphology of fresh and spent catalysts was studied using a transmission electron microscope (TEM) Jeol JEM-2100 (Japan). Particle size distribution and average particle size were calculated by processing at least 800 nanoparticles from TEM images using the Origin Software (USA).

The content of Co and Ru was determined using ICPE-9000 (Japan) inductively coupled plasma emission spectroscopy. Before measurements, 100 g of the sample was treated with a mixture of concentrated acids H_2_SO_4_:HNO_3_ (1:2 molar ratio) for complete dissolution, and hydrofluoric acid was added.

Scanning electron microscopy (SEM) was carried out on a Jeol JIB-4501 instrument (Japan) with an electron tube voltage of 30 kV.

The chemical composition of the surface of the samples was studied using a photoelectron spectrometer from SPECS Surface Nano Analysis GmbH (Germany). The spectrometer is equipped with a PHOIBOS-150-MCD-9 hemispherical analyzer and an XR-50 X-ray characteristic radiation source with a double Al/Mg anode. Nonmonochromatized radiation Al K*α* (h* = 1486.61 eV) was used to record the spectra. To account for the charging effect of the samples, the Si*2p* spectrum of silicon included in the carrier was used. Data processing was performed using the CasaXPS software package. The shape of the peaks was approximated by a symmetric function obtained by multiplying the Gauss and Lorentz functions.

X-ray structural analysis (XRD) of the prepared catalysts was carried out on a Rigaku SmartLab instrument (Japan) with monochromatic Cu K*α*-radiation (k = 1.5418) in the 2θ angle range of 5–80° at a rate of 5 °/min. The identification of the resulting peaks was carried out by comparing the diffraction patterns with a standard library of XRD powder file compiled by the Joint Committee of Powder Diffraction Standards (JCPDS).

The average particle size of Co_3_O_4_ was calculated from the most intense XRD peak (2θ = 36.8°) based on the Scherrer equation (1):

(1) $$dCo_{3}O_{4}=k\lambda /\beta cos\theta$$

where k is a constant, θ is the Bragg diffraction angle, β is the line broadening at half the maximum intensity, λ is the X-ray wavelength.

The average sizes of metallic cobalt in the reduced catalysts and the dispersion were determined using Equations (2) [31] and (3) [32]:

(2) $$do^{0}=0.75*dCo_{3}O_{4}$$

(3) $$\%D=96/do^{0}$$

### 2.4. Catalytic experiment

Catalytic activity was evaluated on a laboratory flow-type Fischer-Tropsch unit with an integral stainless-steel tubular reactor (inner diameter 14 mm) with a fixed bed of catalyst (grain size < 100 μm) mixed with quartz sand (particle size 0.4–1.0 mm) to avoid local overheating. Before testing, the catalysts (0.2 g) were activated in hydrogen flow at 400°C for 4 h. The synthesis of hydrocarbons was carried out with a gradual increase in temperature from 180 to 210°C, CO/H_2_ molar ratio = 1/2, pressure = 2 MPa, gas flow rate = 5 nL/(h × g_cat_). Evaluation of catalytic characteristics (activity, selectivity) was carried out after 56 hours on stream at 210°C after reaching pseudo-stationary conditions.

Light hydrocarbons (C_1_-C_4_) and CO_2_ were analyzed with a gas chromatograph (experimental laboratory unit, Gubkin University-Chromos, based on GC-1000 model, Moscow – Dzerjinsk, Russia) equipped with flame-ionisation detectors (FID) and thermal conductivity detectors (TCD) detectors. Collected liquid hydrocarbons from the trap were analyzed on an experimental laboratory chromatograph (Gubkin University – Chromos, based on GC-1000 model, Moscow – Dzerjinsk, Russia) equipped with an FID detector. The selectivity of C_5+_ was calculated by the difference from the total mass balance and the amount of C_1_-C_4_ and CO_2_ gases.

## 3. Results and discussion

**Scheme 1.**
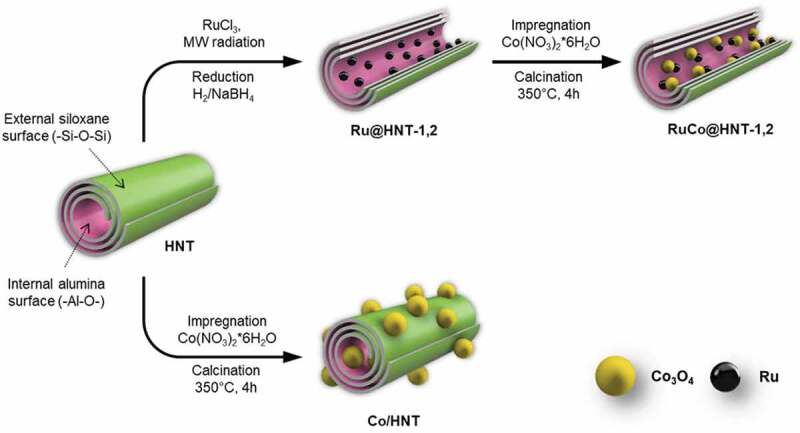
Synthesis of cobalt (Co/HNT) and ruthenium-cobalt (RuCo@HNT-1,2) Fischer-Tropsch catalysts inside aluminosilicate nanotubes.

Formation of bimetallic RuCo nanoparticles inside aluminosilicate nanotubes was achieved by two-step hybrid reduction–impregnation synthesis method (Scheme 1). Microwave radiation (MW radiation) was used to stimulate Ru deposition on the inner surface of clay nanotube at first. This was important because loading of metal salts inside halloysite clay nanotubes with positively charged lumen could not be achieved by simple impregnation [33–35]. After loading, ruthenium salt was reduced with an aqueous solution of NaBH_4_ or in a flow of H_2_ gas at 400°C. At the second stage, a cobalt oxide was added by incipient wetness impregnation. Previously, it was shown that reduction of Ru salt inside clay tubes with NaBH_4_ or in a flow of H_2_ resulted in Ru nanoparticles with different particles size distribution [36]. The reduction with hydrogen gave nanoclusters with size of 1–2 nm, when NaBH_4_ reduction led to formation of larger particles with size of 3–4 nm. To obtain a monometallic cobalt catalyst (Co/HNT), a standard impregnation-calcination method was used [31,37,38].

### 3.1. Physicochemical characteristics

**Figure 1.**
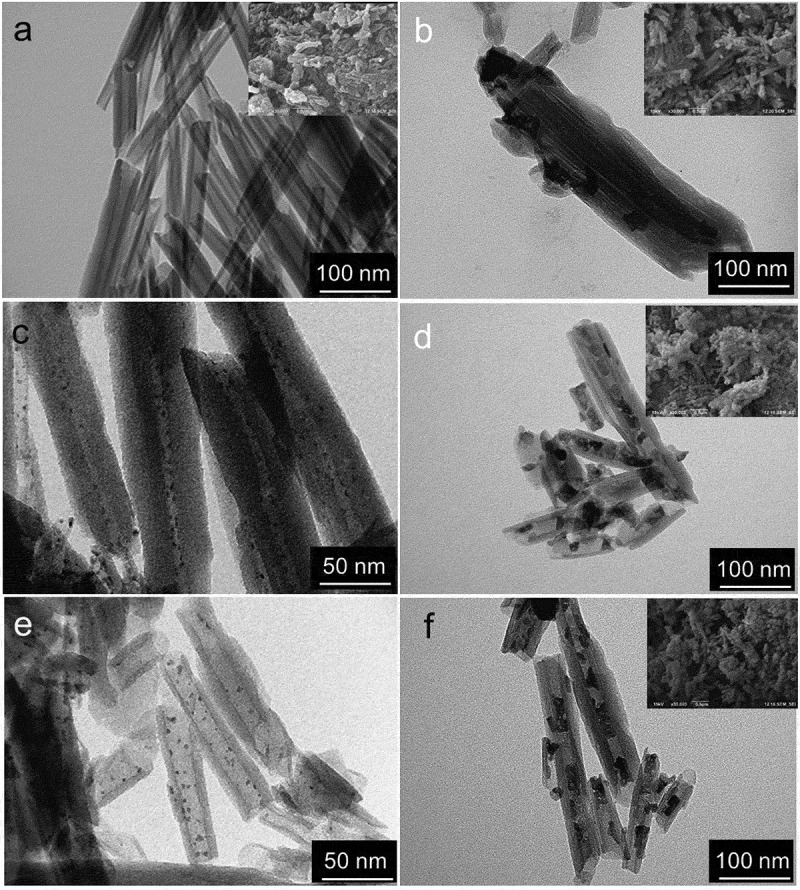
TEM and SEM images of HNT (a) and fresh Co/HNT (b), Ru@HNT-1 (c), RuCo@HNT-1 (d), Ru@HNT-2 (e), RuCo@HNT-2 (f).

The morphology of pure and modified halloysite is shown in Figure 1. Synthesis of monometallic cobalt catalyst (Co/HNT) by impregnation-calcination method resulted in the formation of cobalt oxide nanoparticles both inside and outside the aluminosilicate nanotubes (Figure 1(b)). The developed synthesis technique (Scheme 1) led to the formation of nanoreactors with ruthenium-cobalt oxide nanoparticles located mainly inside halloysite nanotubes (Figure 1(c,d)). The inserts in Figure 1(c,d) are TEM images of nanotubes after first impregnation-reduction step loaded with Ru nanoclusters. Average particle size of Ru nanoclusters in case of reduction with hydrogen was 1.9 nm based on TEM images. In case of reduction of ruthenium salt with NaBH_4_, this value was 2.6 nm. The formation of ruthenium-cobalt oxide selectively inside tubes could be explained by the electron-magnetic action of ruthenium clusters [32].

**Figure 2.**
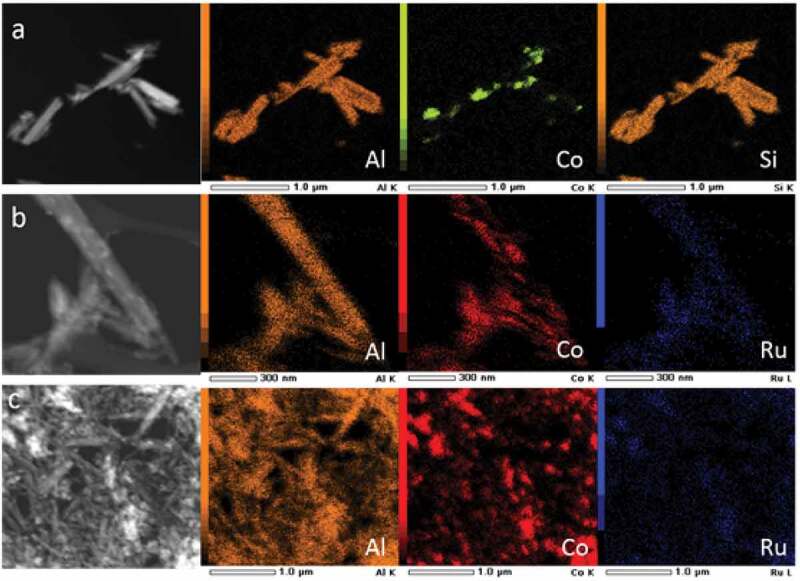
STEM image of Co/HNT (a), RuCo@HNT-1 (b), RuCo@HNT-2 (c) and elemental mapping of correspondent samples showing distribution of atoms within the catalysts.

Scanning transmission electron microscopy (STEM) in combination with energy dispersive X-ray spectroscopy (EDX) was performed to map the distribution of elements in the samples Co/HNT, RuCo@HNT-1, RuCo@HNT-2 (Figure 2). The main elements that make up the structure of the prepared catalysts were Si, Al and Co. In contrast to monometallic cobalt systems for promoted catalysts, a more uniform distribution of cobalt is observed throughout the material with its predominance in nanotubes. Ru position was concluded to be within the tubes; hence, its low concentration makes analysis difficult. Ru and Co positions were coincided.

The cobalt and ruthenium contents were close to the theoretical values in all prepared catalysts according to IСP-ES data (Table 1). Cobalt content in Co/HNT reached 14.5 wt.% (theoretical value was 15 wt.%). In bimetallic catalysts, the amounts of Co and Ru were 14.7 and 14.3 wt.% and 0.12 and 0.15 wt.%, respectively, for RuCo@HNT-1 and RuCo@HNT-2. Both samples contained sodium atoms; in case of RuCo@HNT-1, Na was a part of halloysite structure. Na content in the sample RuCo@HNT-2, Ru nanoparticles in which were obtained by reduction with NaBH_4_, increased from 0.03 to 0.06 wt%. After the Fischer-Tropsch synthesis, a leaching of metals was observed in all catalysts (Table 1). For Co/HNT, decrease in the cobalt concentration reached almost 30 wt%. In case of materials with active particles loaded inside clay nanotubes promoted with ruthenium, the metal leaching varied from 16 to 13 wt% for RuCo@HNT-1 and RuCo@HNT-2. This may be explained by particles stabilization inside halloysite as well as stabilization role of ruthenium.

**Table 1.** Composition of the prepared catalysts before and after the Fischer-Tropsch synthesis

Catalyst	Theoretical		ICP-ES before reaction			ICP-ES after reaction	
	Composition ± 0.01 (wt %)						
	Co	Ru	Co	Ru	Na	Co	Ru
Co/HNT	15.00	-	14.50	-	0.03	10.41	-
RuCo@HNT-1	15.00	0.20	14.70	0.12	0.03	12.29	0.10
RuCo@HNT-2	15.00	0.20	14.30	0.15	0.06	12.40	0.16

The textural properties of halloysite and prepared samples were determined by low-temperature adsorption and desorption of nitrogen (Table 2). For all catalysts, a decrease in surface area, volume and pore diameter was observed as compared to pristine halloysite. The promoted catalysts, in contrast to Co/HNT, had a higher surface area due to a decrease in the particle size of cobalt oxide. This was in accordance with the XRD result [29,39].

**Table 2.** Characteristics and particle size of cobalt and cobalt-ruthenium Fischer-Tropsch catalysts based on halloysite nanotubes

Catalyst	Surface area, BET±1, m^2^/g	Pore volume ±0.01, cm^3^/g	Average pore size, ± 0.1, nm	XRD particles size±0.1, nm	Dispersion ± 0.1, %
HNT	65	0.52	8.0	-	-
Co/HNT	63	0.37	7.2	11.6	8.3
RuCo@HNT-1	59	0.39	7.4	5.6	17.1
RuCo@HNT-2	64	0.42	7.7	4.9	19.6

For all samples, the peaks at 19.2°, 31.5°, 36.8°, 45.8°, 59.4°, 65.3° corresponded to cobalt oxide (Co_3_O_4_) were indicated at XRD patterns [40–42]. Reflection peaks at 12.36°, 20.12°, 25.28° were associated with halloysite clay mineral [43]. CoO was not detected by XRD. Due to the low concentration of the Ru promoter, its presence was not confirmed by X-ray diffraction analysis.

**Figure 3.**
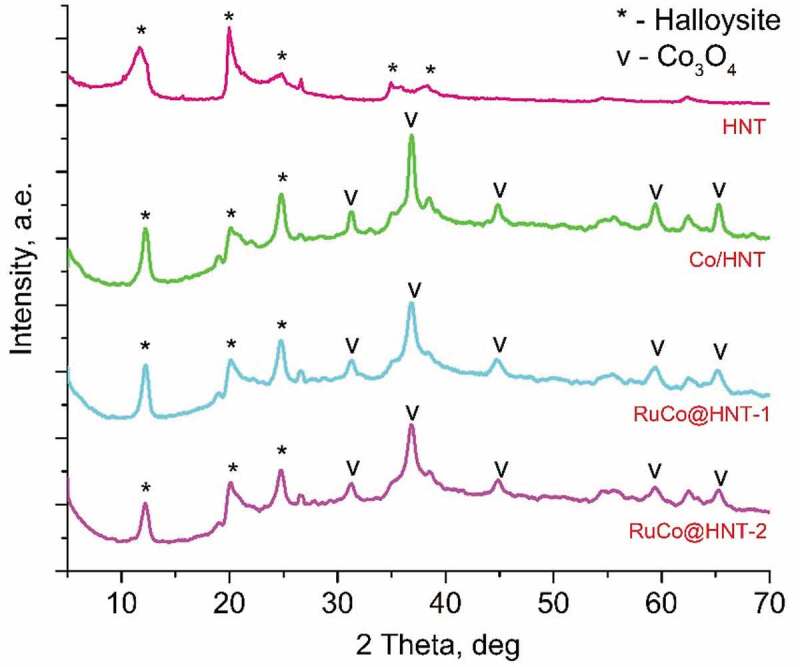
XRD patterns for HNT, Co/HNT, RuCo@HNT-1, RuCo@HNT-2.

The average size of Co_3_O_4_ nanocrystallites was calculated using the Scherrer equation from the most intense peak at 36.8°. Based on the data obtained, the Co crystallite size and metal dispersion were determined for all samples. The dispersion of the particles for RuCo@HNT-1 and RuCo@HNT-2 increased. The presence of ruthenium in catalysts resulted in an increase in the dispersion of cobalt, since small amounts of secondary metal act as an anchor for Co particles and suppress the sintering of cobalt particles during calcination and reduction [28,44]. Kogelbauer et al. [45] reported that the addition of ruthenium reduces the particle size and increases the dispersion of the metal. This statement was confirmed in [46]. Similar results on the effect of ruthenium on the size of cobalt particles were obtained in our work. It should be noted that the dispersion of the RuCo@HNT-2 catalyst was higher than that of RuCo@HNT-1 (Table 2), which can be explained by the promoting effect of boron together with ruthenium [47,48]. (Figure 3)

**Figure 4.**
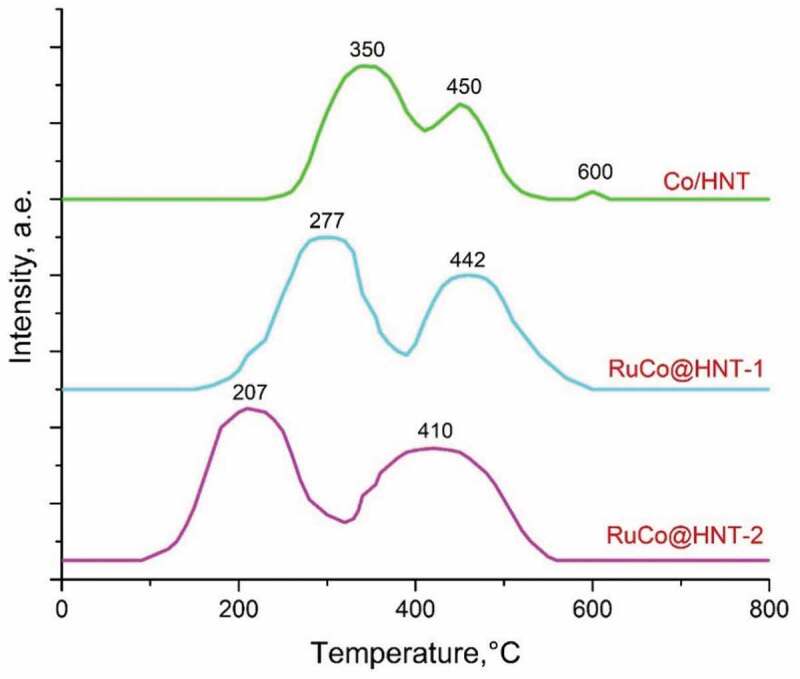
The H_2_-TPR profiles of the Co/HNT, RuCo@HNT-1, RuCo@HNT-2.

Figure 4 shows the H_2_-TPR profiles of the prepared catalysts. All samples are characterized by two main reduction peaks: low-temperature (330–385°C) and high-temperature (400–500°C). The first peak is associated with the reduction of Co_3_O_4_ to CoO by the reaction: Co_3_O_4_ +H_2_ → 3CoO + H_2_O, and the second peak is associated with the transformation CoO + H_2_ → Co + H_2_O [34,49]. Co/HNT has three peaks at 350°C, 450°C, 600°C. The presence of a peak on the TPR curve of Co/HNT at high temperatures (600°C) is associated with a formation of cobalt aluminate [50]. Ruthenium promotion prevents the reaction of cobalt with the carrier, catalyses the reduction of cobalt and inhibits the formation of cobalt aluminates, as evidenced by the disappearance of the high-temperature peak on the TPR-H_2_ curves [7,51]. In contrast to Co/HNT, in case of RuCo@HNT-1 promoted with Ru reduction, peaks were observed at 277°C, 442°C. This result agrees well with the literature and could be explained by hydrogen spillover effect due to the action of a noble metal [7,52]. For example, the bimetallic RuCo catalytic system with 15.0 wt.% of Co and Ru was 0.15 wt.% of Ru obtained by the reduction of a ruthenium salt on Al_2_O_3_, and the followed deposition of cobalt was characterized by reduction peaks at 266°C and 433°C [32].

The shift of the reduction peaks on the H_2_-TPR profile of the RuCo@HNT-2 catalyst to 207°C and 410°C compare to RuCo@HNT-1 was very pronounced. The higher reducibility of RuCo@HNT-2 could be partly explained by the fact that the reduction of smaller particles tends to start at lower temperature. Hence, the promoting effect of boron in synergy with ruthenium should play a significant role [48]. Boron prevented metallic nanoparticles interaction with the support, thus enhancing reducibility. Another reason for the displacement of the temperature peaks of the TPR curves may be related to the inhibitory effect of the chloride ion [53], the content of which may remain after the preparation of RuCo@HNT-1 in the process of obtaining Ru@HNT-1.

**Figure 5.**
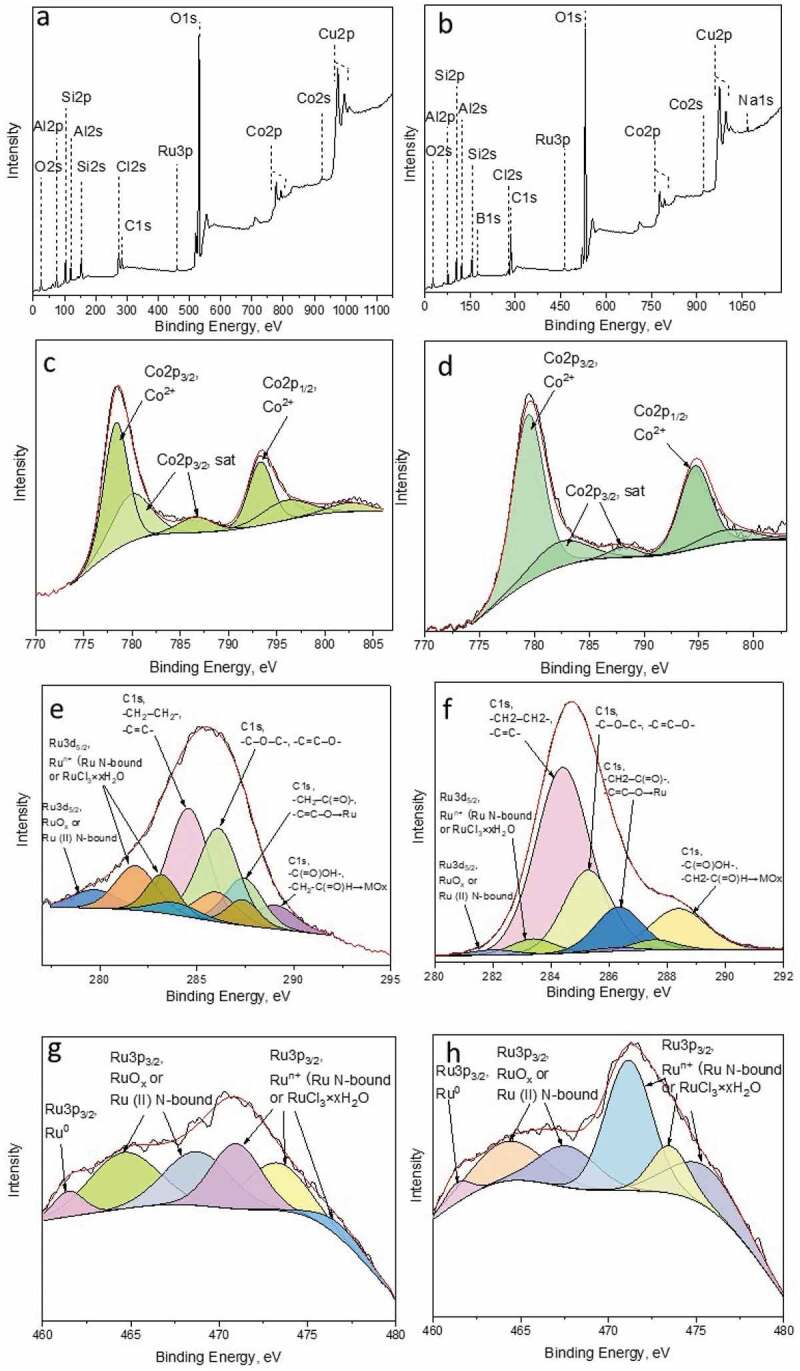
XPS survey spectra of RuCo@HNT-1 (a), RuCo@HNT-2 (b). High-resolution deconvoluted XPS spectra for Co *2p*, Ru*3d+*С*1s* and Ru3*p_3/2_* for RuCo@HNT-1 (c,e,g), RuCo@HNT-2 (d,f,h).

The X-ray photoelectron survey spectra of ruthenium-promoted cobalt catalysts contained different elements (Figure 5(a,b)). Using XPS, it has been proven that boron was present in the RuCo@HNT-2 sample in contrast to RuCo@HNT-1. Na *1s* peak was also detected for RuCo@HNT-2. Chlorine was distinctly visible in XPS spectra of RuCo@HNT-1, and the intensity of Cl 2s peak in case of RuCo@HNT-2 was low.

The Ru*3d* spectrum overlaps with the intense C*1s* spectrum, the main line of which lies in the range 284.7–285.5 eV, which corresponds to carbon in the composition of hydrocarbon impurities (Figure 5(e,f)). The binding energies of Ru*3d_3/2_* are 282.2 and 283.3 eV, which corresponds to Ru in the oxidized state [54,55]. According to the literature data, Ru in the metallic state is characterized by the Ru*3d_5/2_* binding energy in the range of 279.8–280.3 eV. For RuO_2_, the Ru*3d_5/2_* binding energy is 280.5–281.4 eV. For ruthenium compounds, slightly higher binding energies are observed to a greater extent. For example, for RuO_3_ and RuO_4_ in the literature, the Ru*3d_5/2_* binding energies are given in the range of 282.5 and 283.3 eV [55]. In this case, it can be both in the composition of the oxide, for example RuO_2_ with a binding energy of 280.7 eV or RuO_3_ (for higher energies of the order of 283.0–283.5 eV) and in the composition of the RuCl_3_ * xH_2_O salt (281.0–281.5 eV). Ru^0^ species were not identified in the Ru*3d* spectra, probably due to overlapping with C 1s line [56].

In the Ru*3p*_3/2_ spectral region of the catalysts (Figure 5(g,h)), a low-intensity peak was observed at 461.4–461.5 eV, corresponding to Ru^0^. The peaks in the region of 461.5–465.5 eV refer to ruthenium in the composition of the oxide [56] or complex with nitrogen-containing ligands. The presence of peaks in the region of higher energies of 468–477 eV is probably related to ruthenium in a higher oxidation state. These can be both ruthenium and ruthenium oxides in the composition of salts: the hydrated form of ruthenium trichloride or complex compounds, the coordination sphere of which includes nitrogen, sodium, boron, chlorine and organic fragments. For example, Na2 [RuCl_5_ (H_2_O)], Na [Ru (CH_3_C=(O) CHC=(C_6_H_5_) NCH_2_CH (CH_3_) N=C (C_6_H_5_) C=(H) C (O) CH_3_) Cl_2_], Na_2_ [Ru (NO) Cl_5_], Na_2_ [RuCl_5_ (H_2_O)] and others.

It should be noted that, due to the low content of ruthenium in the composition of the samples and the low signal intensity, the spectrum is difficult to interpret. In addition, there are very few data in the literature on the valence state of ruthenium in high oxidation states, and the spread in energies for one and the same form is quite large, which makes it impossible to unambiguously correlate each peak with a specific compound. At the same time, it can be unambiguously concluded that, regardless of the method of reduction, ruthenium is predominantly in an oxidized state.

To identify the state of cobalt, the position of the main Co*2p_3/2_* line, the shape of the Co*2p* spectrum (intensity and relative position of the ‘shake-up’ satellite lines), as well as the magnitude of the spin-orbital splitting of Co*2p_3/2_*–Co*2p_1/2_* were used. The position and intensity of the ‘shake-up’ line of satellites and the magnitude of spin-orbital splitting depend on the chemical state of cobalt and on the chemical environment. In the case of the studied samples, Co*2p_3/2_* represents a symmetrical peak with a binding energy in the range 780.7–781.0 eV, and low-intensity ‘shake-up’ satellites in the range 788–789 eV are also present on the spectrum (Figure 5 (b,c)). There are no intense shake-up satellites in the spectrum of metallic cobalt Co^0^ and Co_3_O_4_ oxide, and the value of the Co*2p*_3/2_ binding energy lies in the range of 778.0–778.2 and 779.5–780.5 eV. Cobalt in the Co^2+^ state is characterized by the binding energies of Co*2p_3/2_* in the range of 780.0–782.0, as well as the presence of an intense (up to 20% of the main peak of Co*2p_3/2_*) shake-up satellite in the range 786–787 eV [57,58]. The value of the binding energy and the presence of a shake-up satellite allows us to say unequivocally that cobalt is in the Co^2+^ state in the studied samples.

**Figure 6.**
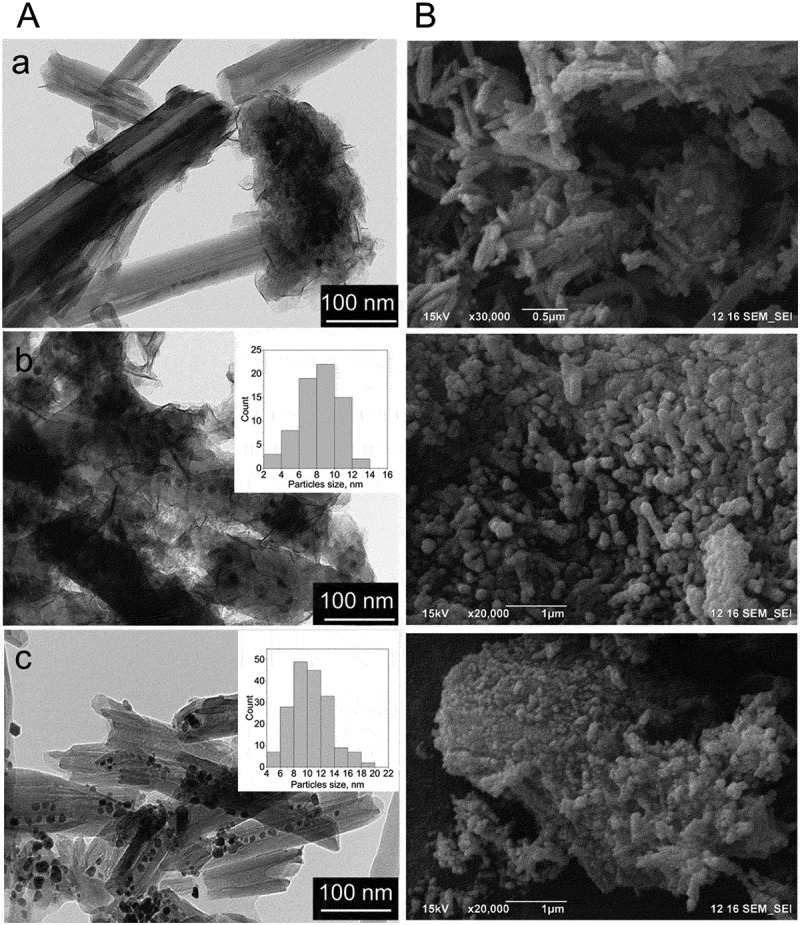
TEM (A) and SEM images (B) of used Co/HNT (a), RuCo@HNT-1 (b), RuCo@HNT-2 (c).

The morphology of nanoreactors after the Fischer-Tropsch synthesis was studied using transmission and scanning microscopes (Figure 6). Co/HNT with Co particles located both on the outer surface and inside tubes was concluded to be unstable (Figure 6(a)). Metal particles underwent significant leaching with the formation of agglomerates with size of 200–700 nm. For the RuCo@HNT-1 and RuCo@HNT-2 systems, no significant agglomeration of metal nanoparticles was observed (Figure 6(b,c)). This was associated with the stabilizing role of the promoter as well as the position of nanoparticles inside tube. It was shown earlier that nanoparticles loaded inside halloysite had much higher stability even in high temperature catalytic processes [59]. The distribution of particles after the Fischer-Tropsch synthesis for both catalysts was narrow and confined by nantoubes’ lumen; the average particle size was 8.2 nm – RuCo@HNT-1 and 9.1 nm – RuCo@HNT-2.

### 3.2. Сatalytic performance

The Fischer-Tropsch synthesis was carried out in a fixed-bed reactor at 210°C. After 32 hours of operation, pseudo-steady-state conditions were reached under which the concentrations of all products at the outlet of the reactor were determined and catalyst performance was evaluated in terms of CO conversion and selectivity to particular products. Also, activity was calculated as the amount of CO converted over 1 mol of Сo per second.

The activity of catalysts increases in the row Co/HNT < RuCo@HNT-1<RuCo@HNT-2 (Table 3). The catalytic activity in FTS depends on both cobalt dispersion and its degree of reduction [39,60]. Under experimental conditions, activation of the catalysts happened in a flow of hydrogen gas at 400°C for 4 hours. As can be seen from the results, the activity of catalysts promoted with Ru differed significantly from Co/HNT. The presence of a promoter contributes to a decrease in the size of cobalt particles in the range lower temperatures, which leads to an increase in reducibility due to the well-known hydrogen spillover mechanism [28]. Small metal ruthenium particles can dissociate hydrogen in the neighbourhood of a supported cobalt particle, leading to the formation of atomic hydrogen that may spill over by diffusion to cobalt. This result in an enhanced degree of cobalt reduction and therefore a higher amount of surface cobalt metal atoms. The result of this promotion is an increase in the number of active sites and therefore a higher activity of promoted catalysts [52]. The lower CO conversion for the RuCo@HNT-1 catalyst as compared to RuCo@HNT-2 can be explained by the lower degree of reduction, which leads to a decrease in the number of available active cobalt sites for carbon monoxide [13]. The lowest activity of Co/HNT, in contrast to RuCo@ HNT-1,2, can be associated with a stronger interaction of Co particles with the support, which leads to the formation of difficult-to-reduce Co aluminate compounds, which negatively affect the Fisher-Tropsch process coefficient [27].

**Table 3.** Performance of the cobalt and cobalt-ruthenium Fischer-Tropsch catalysts based on halloysite nanotubes.^a^

Parameter	Co/HNT	RuCo@HNT-1	RuCo@HNT-2
**СО conversion, %**	7.4	18.4	24.6
**Activity**, **mol_CO_/(s×mol_Co_)×10^3^**	0.61	1.21	1.62
**СН_4_ selectivity, %**	8.1	16.3	11.9
**С_2_-С_4_ selectivity, %**	-	18.5	8.7
**С_5+_ selectivity, %**	90.0	64.4	79.1
**СО_2_ selectivity, %**	-	0.8	0.3
**% olefins in С_2_–С_4_**	–	40.0	42.5
**ASF, α**	–	0.740	0.853

^a^Reaction conditions: P = 2.0 MPa, T = 210°C, H_2_/CO = 2:1, gas flow rate = 5 nL/(h g_cat_), data collected after 56 h on steam

A sharp decrease in CO conversion is observed for the RuCo@HNT-1 catalyst. The decrease in the conversion of materials for the RuCo@HNT-2 catalyst is noticeable in the first 24 hours and almost flattens out at around 25%. Meanwhile, a constant decrease in the degree of CO conversion is characteristic when using a Co/HNT catalyst, and after 56 hours, the conversion is 7.4%. Thus, before reaching pseudo-stationary conditions when using the Co/HNT catalyst, the CO conversion decreased by 9.8%. On cobalt catalysts promoted with ruthenium, feed conversion decreased by 15.6% (RuCo@ HNT-1) and 4.4% (RuCo@HNT-2). Based on the data obtained, we can say about a more stable catalytic activity of RuCo@HNT-2.

**Figure 7.**
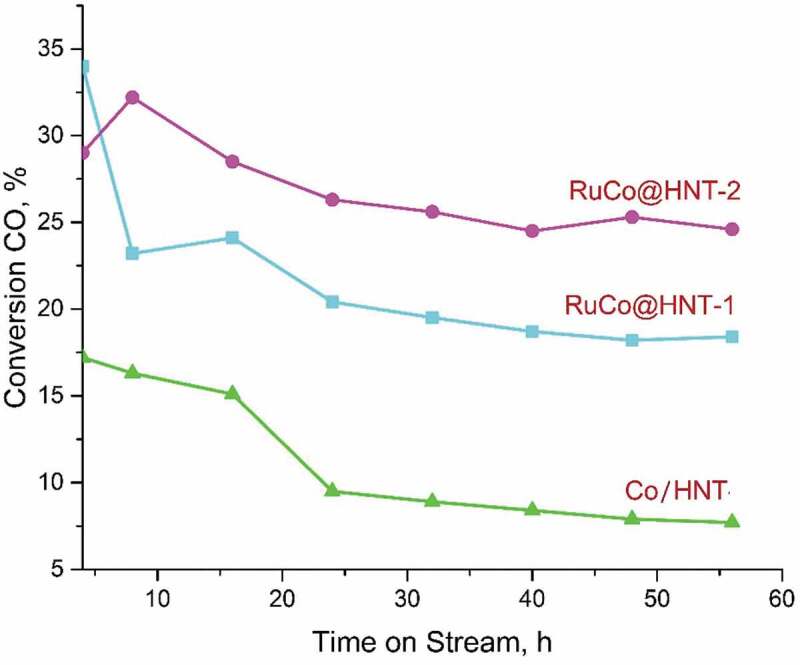
Variation of CO conversion with time on stream for samples Co/HNT, RuCo@HNT-1, RuCo@HNT-2.

The insignificant conversion of CO to CO_2_ indicates a low activity of the catalysts in the water gas shift reaction (Table 3). The synthesis products were only hydrocarbons, and the yield of oxygen-containing compounds was negligible. (Figure 7)

Due to the low activity of Co/HNT, the formation of liquid components was insufficient for the determination of the hydrocarbon composition by the chromatographic method.

**Figure 8.**
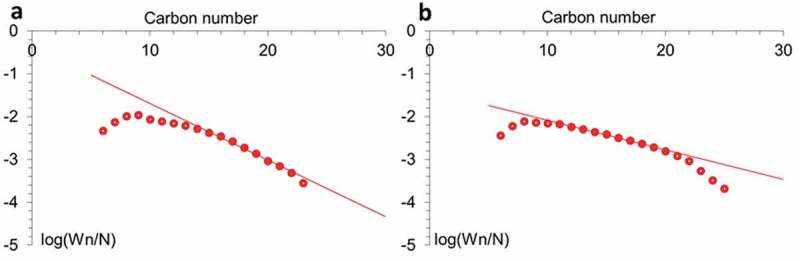
Anderson-Schulz-Flory (ASF) plots of hydrocarbon formation over RuCo@HNT-1 (a), RuCo@HNT-2 (b).

Ruthenium affects both the activity of catalysts and their selectivity. The higher selectivity for C_5+_ found for Ru-promoted catalysts may be due to an increase in the electron density of Co active centers. Apparently, the high density of sites inherent in Co-Ru catalysts leads to enhanced readsorption of α-olefins, which reverses the β-hydrogen abstraction termination step and, thus, favors the formation of higher hydrocarbons [61]. The presence of heavy fractions on promoted catalysts can also be associated with low desorption of products in narrow tubes of halloysite, which leads to their readsorption, continued chain growth reactions (due to this, higher selectivity for C_5+_) and hydrogenation of light olefins [62]. The higher selectivity of RuCo@ HNT-2 was most likely due to the easier dissociation of CO and chain propagation due to the influence of residual sodium acting as an electronic promoter [63,64]. Such additives, as a rule, accelerate reactions associated with the predominant consumption of CO, which leads to the growth of chains, the formation of olefins. Another reason for the higher yield of liquid hydrocarbons can be explained by the distribution of Ru atoms in bimetallic catalyst [65]. The reduction of Ru salt with different reducing agents led to the formation of Ru cluster with different size [36]. In case of Ru@HNT-2, larger nanoclusters were formed (Figure 1(c,d)). The molecular weight distribution of hydrocarbons obeys the Anderson-Schultz-Flory (ASF) formula (Figure 8). The deviation from the Schultz-Flory law for high molecular weight hydrocarbons was associated with incomplete desorption of waxes from the catalyst surface [66]. Some discrepancies with the ASF distribution at low carbon numbers can be explained by the entrainment of light hydrocarbons by the gas stream from the liquid product reservoir.

Bimetallic systems based on aluminosilicate halloysite nanotubes provided significant activity in the hydrogenation of CO, and their selectivity strongly depended on the preparation method. The RuCo@HNT-2 catalyst reduced with aqueous solution of sodium borohydride showed high selectivity towards valuable C_5+_ hydrocarbons containing a large number of heavy fractions.

To prove the efficiency of proposed preparation method and good perspectives of natural clay nanotubes as supports for FTS, the comparative study was conducted based on literature data (Table 4). The bimetallic RuCo catalysts with similar or close composition to the studied system supported on porous silicates, aluminosilicates and carbon nanotubes were reviewed. The catalyst obtained by preliminary precipitation of a ruthenium salt on ɣ-Al_2_O_3_, metal reduction and subsequent impregnation with a solution of crystalline cobalt nitrate hydrate [32] was characterized by lower activity and selectivity to C_5+_ hydrocarbons compared to RuCo@HNT-2. The catalytic system obtained in [29] was also characterized by low values of synthesis gas conversion, аn yield of liquid hydrocarbons and chain growth index. A catalyst with a 0.2% wt. content of Ru and 20% wt. of Co had almost similar CO conversion rate compared to RuCo@HNT-2 and was characterized by a higher chain growth index α = 0.89 [67]. The authors of [68] obtained Fischer-Tropsch catalyst based on a zeolite H-ZSM-5 by joint impregnation of cobalt and ruthenium precursors. As a result, the conversion of CO reached 29%. The selectivity to liquid products was higher when halloysite nanotubes were used as a support for RuCo nanoparticles. In [69], the RuCo catalyst was prepared by joint impregnation of metal salts to MCM-41. According to the data of catalytic tests, the conversion and selectivity to liquid hydrocarbons was not high compare to proposed systems; the chain growth index equaled 0.61. The CoRu/CNT [49] catalyst obtained by sequential impregnation was characterized by a high degree of CO transformation (41.04%), which was better than the results obtained in this work; hence, the selectivity to C_5+_ hydrocarbons was not high in case of CoRu/CNT. SBA-15 was used as a support, and the catalyst based on it was synthesized by the joint impregnation of cobalt and ruthenium precursors with subsequent calcination at 450°C [70]. It was characterized by higher conversion compared to RuCo@HNT-2, but the selectivity for liquid hydrocarbons is slightly lower and is 72.2% [67].

**Table 4.** Comparison of catalytic properties of supported bimetallic RuCo systems with 0.15–1.0 wt.% of Ru and 10–20 wt.% of Co in the Fischer-Tropsch process under moderate temperature (210–220°C)

Ru and Co content, wt.%	Support	Reaction conditions	СО conversion, %	СН_4_ selectivity, %	С_5+_ selectivity, %	α	Ref.
0.15 Ru 15 Co	Halloysite nanotubes	P = 2.0MPa T = 210°C, H_2_/CO = 2:1	24.60	11.90	79.10	0.85	Present work
0.15 Ru 15 Co	ɣ-Al_2_O_3_	P = 15 bar, T = 220°C, H_2_/CO = 2	20.10	20.44	51.49	0.81	[32]
0.15 Ru 15 Co	ɣ-Al_2_O_3_	P = 1 bar, T = 220°C, H_2_/CO = 2	19.00	25.90	46.50	0.72	[29]
0.2 Ru 20 Co	ɣ-Al_2_O_3_	P = 20 bar, T = 220°C, H_2_/CO = 2:1	23.50	5.10	90.70	0.89	[67]
0.3 Ru 17.7 Co	H-ZSM-5	P = 15 bar, T = 240°C, H_2_/CO = 2:1	29.00	15.00	53.00	-	[68]
0.3 Ru 14 Co	MCM-41	P = 10 bar, T = 220°C, H_2_/CO = 2:1	7.70	19.40	33.80	0.61	[69]
1.0 Ru 10 Co	CNT	P = 20 bar, T = 220°C, H_2_/CO = 2:1	41.04	23.40	59.30	-	[49]
0.1 Ru 20 Co	SBA-15	P = 1.0MPa T = 220°C, H_2_/CO = 2:1	34.10	17.20	72.20	-	[70]

## 4. Conclusion

For the first time, bimetallic RuCo nanoparticles with size of 5–6 nm were synthesized inside natural aluminosilicate nanotubes to give nanoreactors active in the Fischer-Tropsch synthesis and selective to high molecular weight hydrocarbons. RuCo nanoparticles were grown inside clay tubes using two-step procedure with Ru nanoclusters loaded at first using microwave irradiation and composing Ru with cobalt by impregnation. It was shown that agents used to reduce ruthenium salt to nanoclusters had a strong influence on the selectivity of resulted catalysts. Catalyst reduced with an aqueous solution of sodium borohydride had much higher selectivity towards valuable C_5+_ hydrocarbons containing a large number of heavy fractions, in comparison with similar systems reduced in a flow of hydrogen. Better reducibility was mostly explained by the promoting effect of boron. Better selectivity was associated with sodium concentration growth in case of sample reduced with NaBH_4_. It has been shown that treatment with hydrogen result in the presence of chlorine atoms that may inhibit reduction and influence activity of catalyst. Proposed nanoreactors were more selective and active in comparison to the systems on the base on synthetic aluminosilicates, silicates, ɣ-Al_2_O_3_, carbon nanotubes. They are based on natural nanoclay and open perspective new materials for the Fischer-Tropsch process.
